# Therapeutic strategies in METex14 skipping mutated non-small cell lung cancer

**DOI:** 10.1186/s13045-021-01138-7

**Published:** 2021-08-23

**Authors:** Leylah M. Drusbosky, Richa Dawar, Estelamari Rodriguez, Chukwuemeka V. Ikpeazu

**Affiliations:** 1 University of Miami Sylvester Comprehensive Cancer Center, 8100 SW 10th Street, Ste 3310F, Plantation, FL 33324 USA; 2 Guardant 360, 505 Penobscot Drive, Redwood City, CA 94063 USA; 3 Division of Medical Oncology, Department of Internal Medicine, University of Miami Miller School of Medicine, 1475 NW 12th Avenue, Miami, FL 33136 USA

**Keywords:** NSCLC, *MET* Exon 14 skipping (*METex14*), Tyrosine kinase inhibitor, Metastasis

## Abstract

METex14 skipping mutations occur in about 3–4% of lung adenocarcinoma patients and 1–2% of patients with other lung cancer histology. The MET receptor tyrosine kinase and its ligand hepatocyte growth factor (HGF) are established oncogenic drivers of NSCLC. A mutation that results in loss of exon 14 in the MET gene leads to dysregulation and inappropriate signaling that is associated with increased responsiveness to MET TKIs. Results from GEOMETRY mono-1 and VISION Phase I/II clinical trials demonstrated significant clinical activity in patients treated with the *MET* Exon 14 skipping mutation inhibitors capmatinib and tepotinib with tolerable toxicity profile. In the GEOMETRY mono-1 trial, capmatinib was especially active in treatment-naïve patients supporting the upfront testing of this oncogenic driver. Tepotinib demonstrated superior activity in the pretreated patients in the VISION trial. Savolitinib is another MET TKI that has shown efficacy in the first- and second-line settings, including patients with aggressive pulmonary sarcomatoid carcinoma. These studies have demonstrated that these TKIs can cross the blood brain barrier and demonstrated some activity toward CNS metastases. *MET* Exon 14 skipping mutation is detected by NGS-based testing of liquid or tissue biopsies, with preference for RNA-based NGS. The activity of capmatinib and tepotinib is limited by the development of acquired resistance. Current research is focused on strategies to overcome resistance and improve the effectiveness of these agents. Our aim is to review the current status of *MET* Exon 14 skipping mutation as it pertains NSCLC.

## Introduction

Comprehensive genomic testing is now standard of care in the management of advanced/metastatic non-small cell lung cancer (NSCLC). Genomic testing identifies common or uncommon actionable genomic alterations that impact therapeutic decision making [1, 2]. The National Comprehensive Cancer Network (NCCN) guidelines recommend testing for certain molecular and immune biomarkers in patients with advanced/metastatic NSCLC to assess eligibility for targeted therapy or immunotherapy [3]. Predictive biomarkers include activating mutations in *EGFR, BRAF, KRASG12C, and ERBB2*, rearrangements *in ALK, ROS1*, *RET*, and *NTRK, MET* amplification or exon 14 skipping mutations, PD-L1 expression, and tumor mutational burden. Therapies targeting these biomarkers have demonstrated greater efficacy when compared to chemotherapy [4–6].

The mesenchymal–epithelial transition (MET) is a tyrosine kinase receptor that is mostly expressed in epithelial cells, whose natural ligand is the hepatocyte growth factor (HGF). MET signaling has been demonstrated to involve cell proliferation, migration, invasion, and survival [7]. Genomic alterations in MET include *MET* exon 14 skipping mutations *(METex14)* or activating mutations, *MET* gene amplification, and MET protein overexpression. However, the presence of *MET* exon 14 skipping mutations is currently, the best-defined predictive biomarker for the use of MET tyrosine kinase inhibitors (TKIs). *MET* exon 14 skipping mutations occur in about 3–4% of patients with adenocarcinomas and in about 1–2% of patients with other NSCLC histology (squamous and sarcomatoid lung cancer) [8]. It appears that this alteration is more frequent in older women who are non-smokers [9]. MET gene amplification which can be due to the increased gene copy number or due to the transcriptional regulation has been detected in many different types of tumors. It has particularly been associated with a mechanism of resistance to EGFR TKIs with low response to MET inhibitors [10]. While coexistence of *MET*ex14 with other oncogenic drivers is not common, *MET*ex14 and MET amplification have been reported together [11]. Both *MET*ex14 and MET amplifications are associated with poor prognosis in patients with NSCLC.

MET TKIs are divided into types I (subtype Ia and Ib), II, and III. Type Ia inhibitors (e.g., crizotinib) block ATP binding to prevent phosphorylation/activation of the receptor; type Ib inhibitors (e.g., capmatinib, tepotinib, savolitinib, AMG 337) are more specific for MET than type Ia inhibitors. Type II inhibitors (e.g., cabozantinib, glesatinib, merestinib) competitively bind a hydrophobic pocket adjacent to the ATP-binding site. Type III (e.g., tivantinib) inhibitors bind allosteric sites rather than the ATP-binding site [12]. Generally speaking, the outcomes of NSCLC patients with *MET* exon 14 skipping treated with currently available therapies are poor. The results from the GEOMETRY mono-1 and VISION trials, respectively, led to the recent regulatory approval of capmatinib, and tepotinib was granted priority review for the treatment of this population of NSCLC patient with advanced disease. These results validate *MET* exon 14 skipping mutations as important oncogenic targets and underscore the need for routine testing by liquid or tissue biopsies.

### Molecular biology of METex14 skip mutation

**Fig. 1:**
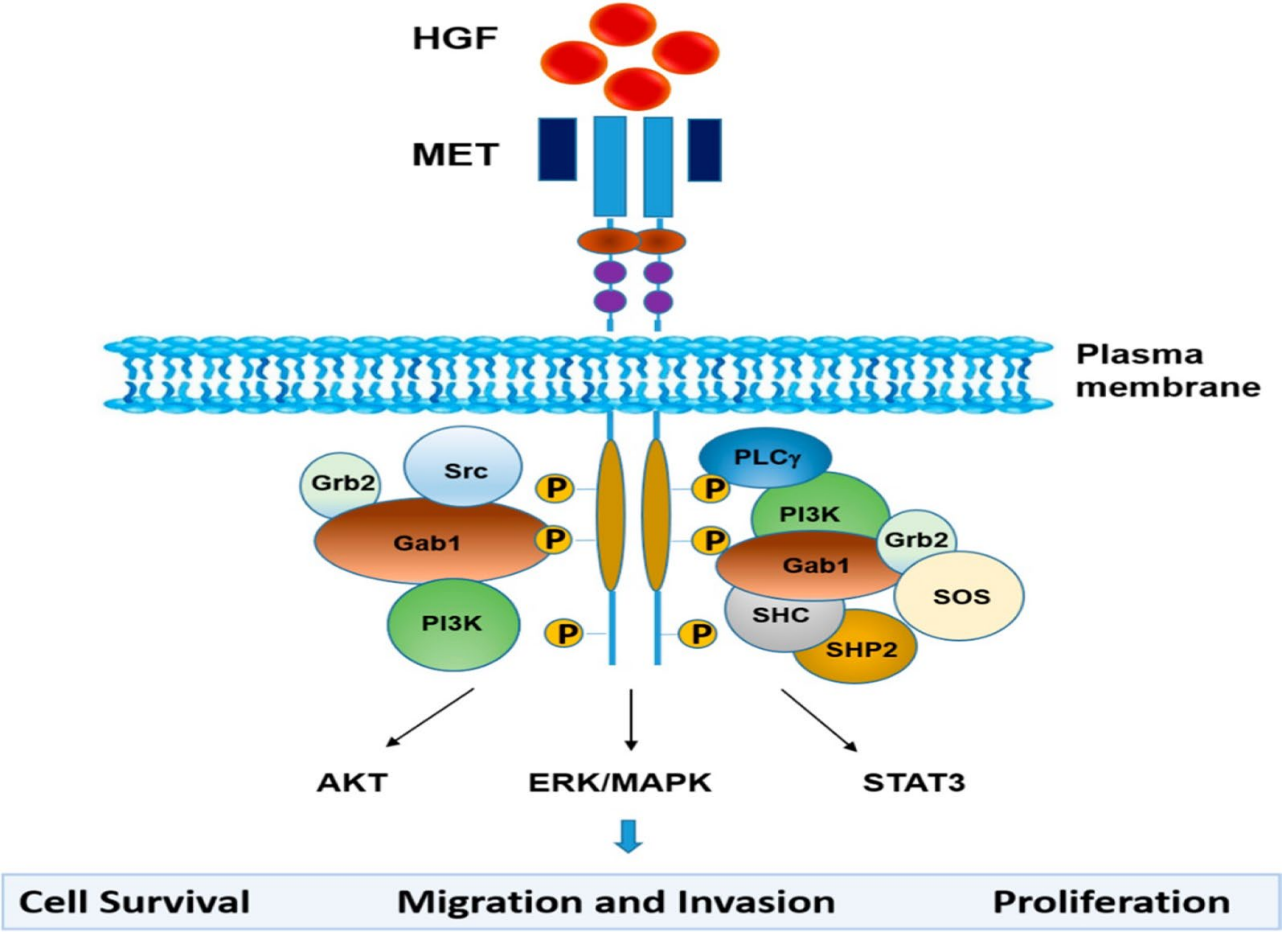
Schematic representation of the HGF/MET signaling pathway [13]. The binding of HGF to MET induces conformational changes, receptor dimerization, trans-phosphorylation of tyrosine residues in the catalytic domain of MET, and phosphorylation of tyrosine residues in the carboxyl-terminal tail. The phosphorylated tyrosine residues create docking sites for several adaptor molecules and kinase substrates. MET activation results in subsequent activation of signaling pathways that include ERK/MAPK, PI3K/AKT and STAT3, which mediate MET-dependent cell proliferation, survival, migration, and invasion. Capmatinib, tepotinib, and savolitinib block the phosphorylation of tyrosine residues

c-MET is known to be expressed in epithelial cells of various organs including pancreas, liver, kidney, prostate, muscle, and bone marrow. When HGF binds to c-MET, the receptor undergoes homodimerization with subsequent phosphorylation of two tyrosine residues Y1234 and Y1235, located in the catalytic loop of the TK domain. Subsequently, Y1349 and Y1356 located within the carboxy-terminal tail also become phosphorylated, forming a tandem SH2 recognition motif. This results in recruitment of signaling effectors, including the adaptor proteins growth factor receptor-bound protein 2 (GRB2), src homology 2 domain-containing (SHC), v-crk sarcoma virus CT10 oncogene homolog (CRK) and CRK-like (CRKL); effector molecules phosphatidylinositol 3-kinase (PI3K), phospholipase Cg (PLCg) and SRC, the src homology 2 domain-containing 5’ inositol phosphatase (SHP2); and the signal transducer and activator of transcription STAT3. Unique to c-MET is its assemblage with GRB2-associated binding protein 1 (GAB1) which is a multi-adaptor protein that creates binding sites for additional downstream receptors upon phosphorylation. GAB1 can either bind directly to c-MET or indirectly via GRB2. Downstream responses of c-MET activation include AKT-mediated cell survival, STAT3-mediated cell proliferation, and ERK/MAPK-mediated cellular migration and invasion [13] (Fig. 1).

Negative regulation of c-MET is necessary for maintaining a tightly controlled activity. The Y1003 site is a negative regulatory site which is located in the juxtamembrane domain that acts by recruiting c-CBL. Regulation of c-MET also occurs via its binding to various protein tyrosine phosphatases (PTPs). These PTPs remove phosphoryl groups on the tyrosines within the c-MET kinase or docking sites. Lastly, binding of PLCg to c-MET activates protein kinase C (PKC) which can negatively regulate receptor phosphorylation and activity. Aside from PKC activation, increases in intracellular calcium levels may also result in the negative regulation of c-MET [14].

*MET* exon 14 skipping mutations result in base substitutions or indels (likely deletions) that disrupt the branch point of intron 13, the 3′ splice site of intron 13, or the 5′ splice site of intron 14 [15]. The region of the protein encoded by exon 14 includes Y1003, which is the binding site of ubiquitin ligase CBL. MET degradation is mediated by ubiquiting ligase CBL. These somatic mutations affect the RNA-dependent splice sites of exon 14 of the gene and activate MET activity via reduction of MET degradation which increases MET stability and activity. There are various molecular variations of *MET* exon 14 skipping alterations, as they exhibit highly diverse sequence compositions, making these mutations difficult to detect. The loss of METex14 results in increased MET stability, prolonged signaling upon HGF stimulation, and increased oncogenic potential. *MET* exon 14 skipping mutations may be may due to genomic deletions ranging in size from a 2-base pair deletion to 193-base pair deletion affecting the splice acceptor or splice donor site, or point mutations involving Y1003 [12].

### Detection of METex14 skipping alterations in tissue and liquid biopsies

*MET* exon 14 skipping alterations are a challenge for diagnostic molecular testing. They exhibit high diversity in sequence composition, many variants are novel, and more than half are indel mutations (up to 3 kb), which are difficult to detect [15]. These variants can be detected by obtaining a tissue biopsy of the tumor and sequencing DNA to identify a variant that alters or ablates a splicing site, or RNA sequencing to observe the direct result of altered splicing [16]. Additionally, *MET* exon 14 skipping mutations can be detected by immunohistochemistry, real-time RT-PCR, and by Sanger sequencing [17, 18]. Utilizing a comprehensive genomic analysis may be the most efficient method to detecting oncogenic driver mutations, including *MET,* since tissue samples are limited. However, not all patients are able to receive comprehensive genomic profiling, and up to 40% of tissue biopsies are not adequate for molecular testing [19, 20].

Liquid biopsies are a well-validated, FDA-approved molecular diagnostic tool that leverage circulating cell-free DNA (cfDNA) shed from advanced-stage solid tumors, which can be interrogated for tumor-specific alterations utilizing hybrid-capture digital next-generation sequencing [21]. Numerous studies have demonstrated the utility of liquid biopsy in identifying oncogenic driver mutations resulting in favorable clinical outcomes when patients are treated with targeted therapy [22–24]. Additionally, liquid biopsy is utilized to detect acquired molecular mechanisms of resistance to targeted therapy, which can be missed if repeated tissue biopsies are not performed at disease progression [23, 25, 26].

### First-line treatment in patients with advanced disease

#### Capmatinib

Capmatinib is a small molecule MET inhibitor which has been shown to effectively inhibit the MET pathway both in vitro and in vivo [27]. In a Phase 1 of study of 43 total patients with advanced, pretreated NSCLC, capmatinib at an established RP2D of 400 mg BID, showed preliminary efficacy with manageable toxicity profile in patients with *MET* exon 14 mutations and c-MET overexpression [28].

GEOMETRY mono-1 was a Phase 2 study that evaluated capmatinib in 364 patients with stage IIIB/IV NSCLC. Patients were assigned to cohorts based on previous lines of therapy and MET status (*MET* exon 14 skipping mutation or *MET* amplification according to gene copy number in tumor tissue). Capmatinib was dosed at 400 mg BID. The study showed that among patients with a *MET* exon 14 skipping mutation, overall response rate was 41% (95% CI: 29–53) of 69 patients who had received one or two lines of therapy previously and in 68% (95% CI: 48–84) of 28 patients who were treatment naive; the median duration of response was 9.7 months (5.6 to 13.0) and 12.6 months (5.6 to NE), respectively (Table 1). The most frequently reported adverse events were peripheral edema (in 51%) and nausea (in 45%); these events were mostly of grade 1 or 2 [29] (Table 2). The study also showed that 54% of patients with brain metastases responded to capmatinib, and 4 patients had a complete response [30]. Based on these data, capmatinib was approved by the FDA for the treatment of adult patients with metastatic NSCLC whose tumors harbor the *MET* exon 14 skipping mutation detected by an FDA-approved test.

**Table 1:** Efficacy of capmatinib in GEOMETRY mono-1

	Previously treated patients (*n* = 69)
ORR (%)	41 (95% CI: 29–53)
Medium DOR (months)	9.7 (95% CI: 5.6–13.0)
Medium PFS (months)	5.4 (95% CI: 5.4–7.0)
	Treatment-naïve patients (*n* = 28)
ORR (%)	68 (95% CI: 48–84)
Medium DOR (months)	12.6 (95% CI: 5.6–NE)
Medium PFS (months)	12.4 (95% CI: 8.2–NE)
	Patients with measurable CNS metastasis (*n* = 13)
CR (%)	31
PR (%)	23

NE denotes not evaluable; ORR denotes objective response rate; DOR denotes duration of response; PFS denotes progression-free survival

**Table 2:** Capmatinib safety overview NSCLC with METex14 skipping mutation (GEOMETRY mono-1)

Adverse events	Cohort 4 (*N* = 69)		Cohort 5b (*N* = 28)	
	Total	Grade 3 or 4	Total	Grade 3 or 4
Any event-no (%)	68 (99)	52 (75)	28 (100)	21 (75)
Peripheral edema	37 (54)	10 (14)	21 (75)	3 (11)
Nausea	31 (46)	0	13 (46)	0
Vomiting	18 (26)	0	7 (25)	0
Creatinine increased	23 (33)	0	10 (36)	0
Dyspnea	19 (28)	7 (10)	6 (21)	2 (7)
fatigue	18 (26)	6 (9)	4 (14)	1 (4)
Decreased appetite	15 (22)	1 (1)	8 (29)	0
Constipation	10 (14)	2 (3)	4 (14)	0
Diarrhea	12 (17)	0	5 (18)	0
Cough	10 (14)	1 (1)	7 (25)	0
Back pain	11 (16)	2 (3)	4 (14)	0
Pyrexia	9 (13)	1 (1)	2 (7)	0
ALT increased	8 (12)	6 (9)	4 (14)	2 (7)
Asthenia	6 (9)	3 (4)	4 (14)	2 (7)
Pneumonia	7 (10)	4 (6)	2 (7)	0
Weight loss	9 (13)	0	3 (11)	0
Non-cardiac chest pain	5 (7)	1 (1)	1 (4)	0
Serious AE	36 (52)	30 (43)	14 (50)	12 (43)
Event leading to discontinuation	14 (20)	8 (12)	6 (21)	5 (18)

Cohort 4 are patients who had received one or two lines of therapy previously and cohort 5b are patients who had not received treatment previously. ALT denotes alanine aminotransferase (30)

#### Tepotinib

Tepotinib is also an oral, ATP-competitive, and highly selective type 1b MET inhibitor [31]. The efficacy and safety of tepotinib have been assessed in a Phase 1 study in patients with solid tumors, including NSCLC. Tepotinib showed antitumor activity, especially in patients with overexpressed or amplified *MET* and was tolerated [31, 32]. The Phase 2 VISION open-label study evaluated tepotinib monotherapy in patients with advanced NSCLC with *MET* exon 14 skipping mutations who were prospectively assessed by liquid and/or tissue biopsy. A total of 152 patients received tepotinib at 500 mg QD and were followed for at least 9 months. The response rate was 46% (95% CI, 36 to 57), with a median duration of response of 11.1 months (95% CI, 7.2 to NE) in the combined biopsy group, by independent review. The response rate was 48% (95% CI, 36 to 61) among 66 patients in the liquid biopsy group and 50% (95% CI, 37 to 63) among 60 patients in the tissue biopsy group; 27 patients had positive results according to both methods. The investigator-assessed response rate of 56% (95% CI, 45 to 66) was similar irrespective of the previous therapy received for advanced or metastatic disease (Table 3). Grade 3 adverse events were seen in 25% of patients, and the most common grade 3 AE was peripheral edema which was seen in 7% of patients (Table 4). Molecular response measured in cfDNA, was observed in 67% of the patients with matched liquid biopsy samples at baseline and during treatment [33]. Based on these data, tepotinib was granted Breakthrough Therapy Designation by the FDA in September 2019 for second-line treatment of patients with metastatic NSCLC harboring *MET* exon 14 skipping mutations who progressed following platinum-based cancer therapy.

**Table 3:** Efficacy of Tepotinib in VISION trial

	Patients with METex14 skipping mutation (efficacy population: *n* = 99)		
	Liquid biopsy (*n* = 66)	Tissue biopsy (*n* = 60)	Combined biopsy (*n* = 99)
ORR (%)	48 (95% CI: 36–61)	50 (95% CI: 37–63)	46 (95% CI: 36–57)
Medium DOR (mo)	9.9 (95% CI: 72–NE)	15.7 (95% CI: 9.7–NE)	11 (95% CI: 7.2–NE)
Medium PFS (mo)	8.5 (95% CI: 5.1–11.0)	11.0 (95% CI: 5.7–17.1)	8.5 (95% CI: 6.7–11.0)

	Patients with brain metastasis (n-11)
ORR (%)	55 (95% CI: 23–83)
DOR (mo)	9.5 (95% CI: 6.6–NE)
PFS (mo)	10.9 (95% CI: 8.0–NE)

NE denotes no evaluable; ORR denotes objective response rate; DOR denotes duration of response; PFS denotes progression-free survival

**Table 4:** Tepotinib safety overview in NSCLC patients with METex14 skipping mutation (VISION Tepotinib trial)

Adverse events-*N* (%)	All grades	Grade 1 or 2	Grade 3
Any	135 (89)	93 (61)	38 (25)
Peripheral edema	96 (63)	85 (56)	11 (7)
Nausea	39 (63)	38 (25)	1 (1)
Diarrhea	33 (22)	32 (21)	1 (1)
Creatinine increased	27 (18)	26 (17)	1 (1)
Hypoalbuminemia	24 (16)	21 (14)	3 (2)
Amylase increased	17 (11)	13 (9)	3 (2)
Lipase increased	13 (9)	9 (6)	4 (3)
Asthenia	12 (8)	11 (7)	1 (1)
Decreased appetite	12 (8)	11 (7)	1 (1)
Pleural effusion	12 (8)	8 (5)	4 (3)
Alopecia	12 (8)	12 (8)	0
Fatigue	11 (7)	10 (7)	1 (1)
ALT increased	11 (7)	7 (5)	3 (2)
AST increased	10 (7)	7 (5)	2 (1)
Vomiting	9 (6)	9 (6)	0
General edema	9 (6)	5 (3)	0
Upper abdominal pain	8 (5)	8 (5)	0

Grade 4 adverse events are not included in the table as there were extremely rare. The listed adverse events occurred in at least 5% of the patients. However, one patient had a combination of respiratory failure and dyspnea related to interstitial lung disease that was reported as a grade 5 adverse event.

The incidence of adverse events of any grade was similar in 39 patients who had received previous immunotherapy and in 113 patients who did not receive such therapy [33].

#### Savolitinib

Savolitinib is a selective oral *MET* TKI and was tested in a Phase 2 single-arm study across 32 hospitals in China and given conditional approval in China for use in patients with NSCLC with *MET* exon 14 skipping alterations (*METex14*) who progressed after previous systemic chemotherapy or unable to receive chemotherapy [34]. Seventy patients with *MET*ex14 skipping alterations were enrolled in this study. The median age of study participants was 68.7 years, 92.9% had stage IV disease, 60% received prior systemic chemotherapy and 35.7% had more aggressive pulmonary sarcomatoid carcinoma (PSC). At baseline, 24.3% of patients had brain metastases.

At a median follow-up of 17·6 months, the IRC-assessed objective response rate was 49·2% in the tumor response evaluable set, and 42.9% in the full analysis. The median PFS was 6.9 months ( with a 50% maturity) and median OS was 14 months. The PFS was reported to be clinically significant in the subgroup of patients with PSC. Treatment-related serious adverse events of grade 3 or more occurred in 32 (46%) patients, the most frequent of which were liver function and peripheral edema. Treatment-related serious adverse events occurred in 27.5% of patients, the most frequent of which were abnormal liver function (4.3%), hypersensitivity reaction (2.9%), and pyrexia (2.9%). Emergence of *FGFR1, EGFR*, and *KRAS* gene amplification at the time of disease progression has been reported as a mechanism of resistance to savolitinib in a case report [35].

### Role of immunotherapy in METex14 skipping alterations

The current NCCN guidelines favor first-line treatment with single-agent targeted therapy for patients with *MET*Ex14 mutation instead of chemotherapy or immune checkpoint inhibitors (ICIs) upfront [1]. Despite the high expression of PDL1 in patients with *MET*Ex14, the efficacy of ICIs in this group is underwhelming. In a large study of 1,387 lung cancer cases, the expression of PDL1 was reportedly high in 49% of MET mutated cases (compared to 29% of MET wild type), while tumor mutation burden was significantly lower in MET mutated cancers compared to wild-type tumors [36]. In a retrospective analysis evaluating immunotherapy activity as monotherapy in advanced NSCLC with oncogenic drivers, the ORR among 36 patients with MET alterations was 16%, with median PFS and OS of 3.4 and 18.4 months, respectively [37]. In another retrospective review, *MET*Ex14 mutations were associated with high (≥ 50%) PD-L1 expression; but the ORR with immunotherapy was low at 17% and median PFS 1.9 months [38]. Other studies have shown conflicting results with similar efficacy of ICIs in MET mutated cancers as wild-type tumors. For example, in a review of twenty-five patients with *MET*ex14 NSCLC, of whom 13 received an immune checkpoint inhibitor in the second-line setting, six patients had prolonged progression-free survival (> 18 months) [39]. Another retrospective multicenter analysis of 30 MET mutated lung cancers confirmed this findings showing an observed response to ICIs of 36%, similar to the non-mutated group [40]. Further prospective data are clearly needed to define the role of ICIs in this distinct subset of oncogene-addicted NSCLC and their potential role in the second-line setting after treatment with a MET TKI upfront.

### Mechanisms of resistance to MET TKIs

MET TKIs have shifted the paradigm in the treatment of patients with *MET* exon 14 skipping mutation. Unfortunately, the response magnitude and duration of response are limited by primary and acquired resistance to MET TKIs. The molecular mechanisms of this resistance are not clearly elucidated.

There have been several established mechanisms of resistance to MET TKIs such as on-target resistance driven by kinase domain mutations affecting drug binding to the receptor or its ATP affinity, amplification of *MET* exon 14 mutant allele, and off-target resistance mediated by the activation of bypass signaling. There are two kinds of MET TKIs: type I and type II MET TKIs based on its binding interaction [41, 42]. Type I MET TKIs (e.g., crizotinib, capmatinib, tepotinib, and savolitinib) bind to MET in its catalytically active conformation where the aspartic acid–phenylalanine–glycine (DFG) motif projects into the ATP-binding site [43–45]. Type II MET TKIs (e.g., cabozantinib, merestinib, and glesatinib) bind to MET in its inactive DFG-out conformation [42, 46]. Type I MET inhibitors can be subdivided into type Ia (crizotinib) or type Ib (capmatinib, tepotinib, and savolitinib) depending on its interaction with the solvent front G1163 residue [41].

MET mutations in residues D1228 and Y1230 confer resistance to type I MET TKI by weakening the chemical bonds between the drug and its receptor [41, 47, 48]. The solvent front mutation G1163R mediates resistance only to crizotinib but not to type Ib or type II MET inhibitors [49]*.* In contrast, mutations in residues L1195 and F1200 confer resistance to type II MET inhibitors [42, 49]. It has also been suggested that resistance to type I MET TKI can be overcome by switching to type II MET inhibitors, particularly if resistance is acquired by mutations involving D1228 and Y1230 residues [42, 49]. Off-target mechanisms of resistance result from bypass track activation of downstream oncogenic signaling in MAPK pathway. Wild-type *KRAS* amplifications, *KRAS* mutations [41, 47, 50], *NF1/RASA1* mutations [51], *PI3KCA* mutations, *EGFR* activation have been shown to drive acquired resistance to MET inhibitors [52, 53]. Acquired *EGFR* amplifications have also been detected in tumor samples from patients whose tumors developed resistance to MET TKIs [41]. Therefore, it is imperative to identify the resistance mechanism to MET TKI by either plasma or tissue next-generation sequencing for the effective targeted treatment of non-small cell lung cancer.

## Discussion

The prognosis with lung cancer remains poor as it remains the leading cause of cancer-related death in the USA. Current therapeutic strategies include traditional chemotherapy, radiation therapy, targeted therapies, and immunotherapies. Actionable mutation within tumors drives the efficacy of targeted therapy. However, tumor heterogeneity remains a challenge for identifying the patient population that may benefit from specific targeted therapy [54].

A meta-analysis of 11 studies with a total of 18,464 patients with NSCLC showed that *ME*T exon 14 skipping mutations were more frequent in women than men, were less likely to be associated with a history of smoking, and were associated with a significantly older age. *MET* exon 14 skipping mutation have also been associated with poor prognosis, but were not associated with an increased risk for stage IV disease [55, 56].

The *MET* exon 14 skipping mutation inhibitors capmatinib, tepotinib, and savolitinib have been proposed for the treatment of adult patients with metastatic NSCLC whose tumors harbor this mutation, irrespective of tumor histology. These TKIs have demonstrated durable response in both untreated and pretreated patients. In treatment-naïve patients, capmatinib demonstrated ORR of 68%, disease control rate of 96.4%, and duration of response of 12.6 months. In previously treated patients, the ORR is 41%, disease control rate is 78.3%, and duration of response is 9.7 months. Thirteen evaluable patients in the cohorts with *MET* exon 14 skipping had brain metastasis at baseline [57] and intracranial response was 54%. There were 31% complete responses and 23% partial responses. Survival data are pending.

In the tepotinib VISION trial, at a median follow-up of 17.4 months, oral tepotinib led to an independently assessed objective response rate (ORR) of 46.5% among the 99 participants with locally advanced or metastatic disease who had been followed up for a minimum of 9 months at data cutoff. There were no complete responses, as all responses were partial and lasted for a median of 11.1 months. Median progression-free survival was 8.5 months and the overall survival data were not mature. In all, 43 patients were treatment-naïve, while the remaining 56 had received at least one prior line of therapy, but the ORRs by independent review were comparable to that of the overall cohort, at 44.2% and 48.2%, respectively. This was also the case for most other subgroups, with the largest difference seen for patients with versus without a smoking history, for whom the respective ORRs were 56.5% and 35.6% [48].

Despite the durable responses seen with these agents, acquired resistance remains a challenge as in all TKIs. Acquisition or emergence of preexisting clones with mutations in the MET activation loop Y1230 (also known as Y1248) or D1228 (also known as Y1246) has been shown to mediate resistance to type I MET TKI such as crizotinib in NSCLC with *MET* exon 14 skipping mutation. However, sensitivity to type II kinase inhibitors such as cabozantinib is maintained, thereby providing the rational for sequential therapy [58, 59]. Increased expression of transforming growth factor α with resultant activation of the EGFR pathway is another cause of resistance [60]. Drug switching and/or combination therapy may be required to target resistance to MET TKIs.

A multitude of new agents and rational combination of agents with *MET* exon 14 skipping as target are currently undergoing clinical trials (Table 5). A randomized Phase II trial evaluating the combination of capmatinib with spartalizumab immunotherapy compared to capmatinib alone in treatment-naïve NSCLC harboring *MET* exon 14 skipping is currently enrolling in Europe and Japan. Antibody–drug conjugates like telisotuzumab vedotin (ABBV-399), which is a first-in-class conjugate of a MET antibody, ABT-700, and the antimicrotibule agent momomethyl auristatin E have been proposed. Other highly selective MET inhibitors like glumetinib have shown robust antitumor activity in preclinical models and are currently being studied in Phase I/II trials. Boxitinib (APL-101) is also a selective MET inhibitor under investigation in Phase I/II trials as a single agent in patients harboring *MET* exon 14 skipping mutation (Table 5). A search of clinicaltrials.gov does not reveal any ongoing trials of *MET* Exon 14 skipping mutation inhibitors in the small cell lung cancer space.

**Table 5:** Ongoing MET inhibitor clinical trials [61]

ClinicalTrials.gov identifier	Study agent	Trial description
NCT02609776	Amivantamab	A Phase 1, First-in-Human, Open-Label, Dose Escalation Study of JNJ-61186372, a Human Bispecific EGFR and c-MET Antibody, in Subjects With Advanced Non-Small Cell Lung Cancer
NCT03175224	APL-101	Phase 1/2 Multicenter Study of the Safety, Pharmacokinetics, and Preliminary Efficacy of APL-101 in Subjects With Non-Small Cell Lung Cancer With cMETex14 Skip Mutations and cMET Dysregulation Advanced Solid Tumors
NCT01639508	Cabozantinib	A Phase II Study of Cabozantinib in Patients With RET Fusion-Positive Advanced Non-Small Cell Lung Cancer and Those With Other Genotypes ROS1 or NTRK Fusions or Increased Met or AXL Activity
NCT02414139	Capmatinib	A Phase II, Multicenter Study of Oral c-MET Inhibitor INC280 in Adult Patients With EGFR Wild-type (wt), Advanced Non-small Cell Lung Cancer (NSCLC)(Geometry Mono-1)
NCT04270591	Glumetinib	A Phase Ib/II, Open-Label, Multicenter Study to Evaluate the Efficacy and Safety of Glumetinib (SCC244), a Selective MET Inhibitor in Patients With Advanced Non-Small Cell Lung Cancer Harboring MET alterations
NCT03539536	Telisotuzumab vedotin	Phase 2, Open-Label Safety and Efficacy Study of Telisotuzumab Vedotin (ABBV-399) in Subjects With Previously Treated c-Met + Non-Small Cell Lung Cancer
NCT02864992	Tepotinib	A Phase II Single-arm Trial to Investigate Tepotinib in Advanced (Locally Advanced or Metastatic) Non-Small Cell Lung Cancer with METex14 (METex14) Skipping Alterations or MET Amplification (VISION)
NCT03993873	TPX-0022	Phase 1 Study of TPX-0022, a MET/CSF1R/SRC Inhibitor, in Patients With Advanced Solid Tumors Harboring Genetic Alterations in MET
NCT04077099	REGN5093	A Phase 1/2 Study of REGN5093 in Patients with MET-Altered Advanced Non-Small Cell Lung Cancer
NCT04323436	Capmatinib	A Double-blind, Placebo-Controlled, Randomized, Phase II Study Evaluating the Efficacy and Safety of Capmatinib and Spartalizumab vs Capmatinib and Placebo as 1^st^ Line Treatment for Advanced NSCLC Patients With METex14 Skipping Mutation

The molecular biology and therapeutic implications of MET alterations in NSCLC continue to evolve. Genomic alterations in MET include *MET* exon 14 skipping mutations or activating mutations, MET gene amplification, and MET protein overexpression, but the presence of *MET* exon 14 skipping mutations is presently the best-defined oncogenic driver and predictive biomarker for the use of MET TKIs. Given the prevalence of *MET* exon 14 skipping mutations and the poor outcomes in these patients, *MET* Exon 14 skipping mutations will continue to be an attractive therapeutic target. As far as we know, *MET* Exon 14 skipping mutation has not been found in small cell lung cancer.

## Conclusion

In conclusion, in the era of precision medicine, it is imperative to evaluate each patient individually. Molecular profiling of the tumor is an essential component of this clinical evaluation process. MET inhibitors are now established TKIs in the treatment of NSCLC patients with exon 14 skipping mutation, which is present in 3–4% of patients with adenocarcinomas and 1–2% of patients with other histologies. The challenge remains overcoming resistance to these new agents.

## Data Availability

Not applicable as no datasets were generated or analyzed.

## Abbreviations

TKI: Tyrosine kinase inhibitor
ALK: Anaplastic lymphoma kinase
ROS1: ROS proto-oncogene 1
PIK3CA: Phosphatidylinositol-4,5-bisphosphate 3-kinase, catalytic subunit alpha
MET: MET proto-oncogene, receptor tyrosine kinase
BRAF: BRaf proto-oncogene
EGFR: Epidermal growth factor receptor
NTRK: Neurotrophic tyrosine receptor kinase
RET: RET Proto-oncogene
KIF5B: Kinesin family member 5B
CCDC6: Coiled-coil domain-containing protein 6
NCOA4: Nuclear receptor coactivator 4
AKT: AKR mouse thymoma kinase
MAPK: Mitogen-activated protein kinase
JNK: C-Jun N-terminal kinase
MEN 2: Multiple endocrine neoplasia 2
MKI: Multi-kinase inhibitor
NGS: Next-generation sequencing
cfDNA: Cell-free DNA
RTK: Receptor tyrosine kinase
RECIST: Response evaluation criteria in solid tumors
ICI: Immune checkpoint inhibitor
ORR: Objective response rate
PFS: Progression-free survival
OS: Overall survival
DCR: Disease control rate
DOR: Duration of response
NCCN: National Comprehensive Cancer Network
